# Accurate flux predictions using tissue-specific gene expression in plant metabolic modeling

**DOI:** 10.1093/bioinformatics/btad186

**Published:** 2023-04-11

**Authors:** Joshua A M Kaste, Yair Shachar-Hill

**Affiliations:** Department of Biochemistry and Molecular Biology, Michigan State University, 603 Wilson Rd., East Lansing, Michigan 48824, United States; Department of Plant Biology, Michigan State University, 612 Wilson Rd., East Lansing, Michigan 48824, United States; Department of Plant Biology, Michigan State University, 612 Wilson Rd., East Lansing, Michigan 48824, United States

## Abstract

**Motivation:**

The accurate prediction of complex phenotypes such as metabolic fluxes in living systems is a grand challenge for systems biology and central to efficiently identifying biotechnological interventions that can address pressing industrial needs. The application of gene expression data to improve the accuracy of metabolic flux predictions using mechanistic modeling methods such as flux balance analysis (FBA) has not been previously demonstrated in multi-tissue systems, despite their biotechnological importance. We hypothesized that a method for generating metabolic flux predictions informed by relative expression levels between tissues would improve prediction accuracy.

**Results:**

Relative gene expression levels derived from multiple transcriptomic and proteomic datasets were integrated into FBA predictions of a multi-tissue, diel model of *Arabidopsis thaliana*’s central metabolism. This integration dramatically improved the agreement of flux predictions with experimentally based flux maps from ^13^C metabolic flux analysis compared with a standard parsimonious FBA approach. Disagreement between FBA predictions and MFA flux maps was measured using weighted averaged percent error values, and for parsimonious FBA this was169%–180% for high light conditions and 94%–103% for low light conditions, depending on the gene expression dataset used. This fell to 10%-13% and 9%-11% upon incorporating expression data into the modeling process, which also substantially altered the predicted carbon and energy economy of the plant.

**Availability and implementation:**

Code and data generated as part of this study are available from https://github.com/Gibberella/ArabidopsisGeneExpressionWeights.

## 1 Introduction

A grand challenge for systems biology is the ability to accurately predict complex phenotypes from omic datasets based on functional principles and mechanisms. Patterns of cellular metabolism—flux maps—are one such complex phenotype (Ratcliffe and Shachar-Hill 2006), for which tools to predict phenotypes from basic assumptions have proven useful in exploring and designing metabolic capabilities (Burgard et al. 2003; Orth et al. 2010; Chen et al. 2011). Methods to quantify flux maps from labeling data now allow the testing of such predictions in both simpler and multicellular systems. However, the integration of omic data to improve the accuracy of flux predictions is still at an early stage.

Metabolic flux predictions are also important for real-world applications since modifying an organism’s metabolic activity in order to achieve some practical aim, such as overproducing a specific metabolite, is central to many biotechnology projects. As in other areas of engineering, metabolic engineering can benefit from mathematical models that describe and predict the behavior of the relevant system(s). Researchers have developed two major modeling approaches to address this need: (i) ^13^C-metabolic flux analysis (^13^C-MFA) and (ii) flux balance analysis (FBA; Orth et al. 2010; Antoniewicz 2015). With ^13^C-MFA, steady-state or kinetic isotopic labeling data for metabolites in a small- to medium-sized network are used to obtain estimates of the net and exchange fluxes through that network (Antoniewicz 2015). These metabolic flux maps are regarded as the most reliable measures of *in vivo* metabolic fluxes; however, the throughput of this technique is limited by the large amounts of isotopic labeling data and other measurements needed to generate each flux map. FBA, which is based on applying conservation principles to a network of reactions using one or more assumptions about the functional objective(s) driving biological organization, requires substantially less experimental input data and is therefore an attractive and commonly used metabolic modeling technique.

FBA and related metabolic modeling methods in microbial systems, together with genome-scale models (GEMs) that represent the biochemical reactions encoded in an organism’s genome, have enabled radical modification of microbial central metabolism (e.g. Gleizer et al. 2019) and substantial improvements in bioproduct yields (e.g. Park et al. 2007, Lee et al. 2007). These methods can, e.g. allow bioengineers to predict the behavior of their system and identify interventions, such as gene knock-outs or knock-ins, that will help them modify the organism’s phenotype (Burgard et al. 2003, Tepper and Shlomi 2009). However, many metabolic engineering applications require the modification not of microorganisms, but of multicellular eukaryotes like plants. Most GEMs of plants to date (e.g. Poolman et al. 2009; Dal’Molin et al. 2010ab; Saha et al. 2011; Arnold and Nikoloski 2014), have treated plants, which are composed of multiple tissues with substantial functional diversity, as single-tissue aggregated metabolic networks. This has motivated the creation of “multi-tissue” GEMs to investigate source-sink dynamics and resource allocation, with the earliest efforts in this space focusing on the interplay between mesophyll and bundle-sheath cells in C4 photosynthesis (Dal’Molin et al. 2010b; Shaw and Cheung 2020).

Re-engineering of plant metabolism on the scale seen in microbial systems has not, to date, been possible and predictive modeling has been neither validated in detail nor applied to successful plant metabolic engineering. This is partly due to the ease and high throughput of microbial transformation relative even to model plant systems*.* In addition to the greater experimental demands, the metabolic modeling of these systems is also substantially harder. There is, consequently, a relative lack of MFA datasets with which to compare the predicted flux maps from FBA in plants. This contrasts with the availability of rich multiomic datasets combining flux estimates with transcript and protein data for a number of different genotypes and growth conditions in systems like *Escherichiacoli* (Ishii et al. 2007). The challenges involved in generating ^13^C-MFA flux maps for plants make improvement of plant FBA flux predictions an attractive path towards replicating the biotechnological successes seen in microbes.

An appealing approach to improving the quality of plant FBA predictions is the integration of additional network-wide data from transcriptomic and proteomic datasets. Gene expression data—particularly transcript data—are substantially easier to generate than ^13^C-MFA flux maps. Previous attempts at integrating gene expression datasets into metabolic flux predictions have been reviewed elsewhere (Machado and Herrgård 2014; Vijayakumar et al. 2017). Methods developed before 2014 were evaluated on the basis of their ability to improve upon parsimonious FBA (pFBA; Lewis et al. 2010) in terms of their predictions’ agreement with MFA-estimated fluxes in microorganisms and were found to not do so reliably (Machado and Herrgård 2014). A key limitation of these studies was a lack of comparison of FBA predictions against ^13^C-MFA-derived flux estimates. This lack of comparison against ^13^C-MFA is shared by the plant FBA literature, in which we are aware of only a small number of evaluations under heterotrophic conditions in green algae (Boyle et al. 2017), *Arabidopsis* cell cultures (Williams et al. 2010; Cheung et al. 2013), and *Brassica napus* embryos (Hay and Schwender 2011). Since then, several studies have developed algorithms benchmarked by their ability to make predictions in agreement with empirical flux maps derived from MFA studies (Tian and Reed 2018; Pandey et al. 2019; Ravi and Gunawan 2021). These studies have focused on unicellular organisms or animal tissues modeled in isolation. Their application to FBA in more complex systems is limited by the large number of resource-intensive MFA datasets needed to calibrate them (Tian and Reed 2018) or their need for a reference expression dataset paired with an assumed-correct flux map (Pandey et al. 2019; Ravi and Gunawan 2021).

To improve the accuracy of FBA in multicellular systems, particularly plants with their complex metabolic networks, we developed a method that integrates tissue-atlas data from multi-tissue systems into the flux-minimization procedure employed in pFBA. This method incorporates evidence from gene expression datasets into FBA metabolic flux predictions by applying weights to individual reactions according to the relative transcript or protein expression of the gene(s) assigned to those reactions between different modeled tissues. The method is evaluated on its ability to make predictions in agreement with MFA flux maps. We demonstrate substantial improvements in the agreement of our FBA-predicted fluxes with flux estimates from a ^13^C-MFA study on *Arabidopsis thaliana* rosette leaf central metabolism (Ma et al. 2014). Finally, we show that multiple gene expression datasets, when used as inputs, result in similar improvements in agreement and that this result generalizes across different MFA flux maps. This approach has particular potential for plant and animal systems for which there are only a limited number of experimental flux maps.

## 2 Materials and methods

### 2.1 Overview of approach

Our method makes two key assumptions: (i) Metabolic flux maps predicted from pFBA (Lewis et al. 2010), minimizing the sum total of flux through the network, are more likely to reflect real flux maps than ones not subject to this constraint, and (ii) A reaction present in two tissues *A* and *B* catalyzed by an enzyme encoded by a gene that is highly expressed in *A* and poorly expressed in *B* is likely to carry higher flux in tissue *A*.

We incorporate assumption 1 by making the objective function of our FBA optimization the minimization of total flux, the same as pFBA (Lewis et al. 2010). This is represented mathematically as finding the minimum value of the linear combination of all fluxes in the network, with each flux v_i_ multiplied by a corresponding coefficient *c_i_*:


(1) min∑j∈Reactionscj*vj

Where *Reactions* is the list of all reactions *j* in the network, *v_j_* is the flux through a reaction *j*, and c_j_ is the coefficient—hereafter referred to as a *penalty weight* since it represents a penalization of the likelihood of using a reaction *j* to carrying flux. When *c_j_* takes a value of 1 for all reactions, our method reduces to pFBA, which can be seen as the limiting case of gene expression having no influence in predicting network flux patterns. We incorporate assumption 2 by calculating, for each reaction in our network model, a coefficient derived from the relative expression of genes encoding the enzyme(s) that catalyze that reaction between the different tissues in the gene expression dataset. The association between reactions and genes is captured by the gene-protein-reaction (GPR) terms in the model. This results in reactions mapped to relatively highly expressed genes receiving small values of *c_j_* and reactions mapped to minimally expressed genes receiving large ones. This use of the coefficient vector to account for relative expression evidence is related to the approach taken in Jenior et al. (2020). However, among other differences in implementation, the two methods differ in their assumed relationship between gene expression and flux and their application. Our method compares gene expression across tissues within a multi-tissue model to generate more accurate flux predictions, rather than comparing the expression of genes to the most expressed gene in a dataset as a proxy for transcriptional investment and a way of generating context-specific models.

### 2.2 Model construction and dataset selection

The *A.thaliana* core metabolism model developed in Arnold and Nikoloski (2014) was used as the basis for a multi-tissue diel model. This model was chosen due to its rich GPR annotation and focus on central metabolism. The core model was duplicated six times to create leaf, stem, and root versions of the model for both day and night, which were interconnected by transporters allowing the movement of specific compounds and metabolites. The substrates, products, and constraints applied to the model can be found in the Supplementary Methods. The model used in this study can be found in Supplementary Dataset S2.

^13^C-MFA flux maps were obtained *in planta* in *A.thaliana* by Ma et al. (2014), and these were used as the empirical best estimates of flux distributions. Although there are not any other ^13^C-MFA flux maps available of autotrophic *A. thaliana* leaves Szecowka et al. (2013) provide estimates of select fluxes in autotrophic *A. thaliana* leaf central metabolism, which we used for additional confirmation of our method’s efficacy*.* The pairing of fluxes in both flux studies to the FBA network is described in Supplemental Dataset S1.

We searched the literature for high-quality, high-coverage RNA-seq, and quantitative proteomic tissue atlases and found two suitable datasets meeting these criteria: Klepikova et al. (2016) and Mergner et al. (2020). The proteomic dataset from Mergner et al. (2020) is a mass spectrometry-based quantitative proteome that reports IBAQ (Intensity-Based Absolute Quantification) values, which are an accurate measure of protein abundances (Krey et al. 2014). For bioinformatic processing details, see Supplementary Methods. For dataset IDs, growth conditions, and key parameters from each study, see Supplementary Tables S4 and S5.

### 2.3 Penalty weight vector calculation

We calculated the expression weight for each gene in each tissue on the basis of how the expression of a reaction in a particular tissue, as measured by transcriptomic or proteomic abundance, compared with the expression of that same gene in the other tissues.


(2) $$W_{it}=\;\frac{\mathrm{Max}{(E}_{i})}{E_{it}}$$

where *W*_it_ is the expression weight for a given gene *i* in a tissue *t*, *E_i_* is the list of expression values of gene *i* for each tissue, *E_it_* is the expression of gene *i* in tissue *t*, and Max() is the maximum value from a set of one or more elements. Note that although the transcriptomic and proteomic datasets used in this study report absolute quantities, our method is applicable as long as relative amounts of RNA or protein across tissues are available. Many GPRs in the model consist of multiple genes that represent isozymes or members of protein complexes. The former are denoted by OR terms and the latter by AND terms in the GPR formulation. This results in many reactions having more than one expression weight due to being mapped to multiple genes. We combine these multiple weights into a single penalty weight value for each reaction by averaging the expression weights of isozymes and taking the “worst” (i.e. largest, most penalizing value) when genes form subunits of a protein complex. As an example, the penalty weight for a reaction *R* in the leaf subnetwork of our model with a GPR of the form (Gene1 OR Gene2) AND (Gene3), corresponding to a protein complex made of the product of Gene 3 and the product of Genes 1 or 2, would be represented by:


(3) cR,lf=SF  MaxWgene1,lf+Wgene2,lf2, Wgene3,lf-1+1

where *c_R_*_,lf_ represents the overall penalty weight in the leaf (lf) for reaction *R*, SF (or the scaling factor) is a coefficient that modulates the magnitude of the calculated penalty weights and *W_gene1_*_,lf_, *W_gene2_*_,lf_, and *W_gene3_*_,lf_ are the penalty weights for the individual genes *Gene1*, *Gene2*, and *Gene3*. Note that in the present implementation of this method, stoichiometric coefficients in GPR terms are ignored. When one or more genes contained in a GPR for a reaction/tissue combination are all more highly expressed than the same genes in the other tissues, the scale for that reaction/tissue combination will be 1. For reaction/tissue combinations that have no corresponding GPR, we explored setting the penalty weights to 1 or a value calculated from the median penalty weight assigned to reactions in the same tissue (for details, see Supplementary Methods).

### 2.4 Optimization

The optimization performed in this paper is a variation on pFBA, which finds the flux map(s) satisfying imposed constraints with minimum total flux through the network (Lewis et al. 2010). The minimization of total flux (Equation 1) is subject to the following constraints:


(4) S*v=0

(5) $$LB_{j}\leq v_{j}\leq UB_{j}$$

(6) $${v_{\mathrm{biomass}}}_{\left(\mathrm{tissue}\right)}=v_{\mathrm{fixed}\;\mathrm{biomas}s_{\mathrm{tissue}}}$$

Where *S* is the stoichiometric matrix of the metabolic network being modeled, *v* is the vector of all fluxes, LB and UB are the vectors of all upper and lower bound constraints, and *v*_biomass(tissue)_ and *v*_fixed biomass(tissue)_ are the biomass flux for a given tissue and the defined biomass constraint for that tissue, respectively. Equation (4) represents the steady state of all internal metabolites, Equation (5) represents the bounds and reversibility constraints, and Equation (6) represents the definition of biomass accumulation rates. All optimizations were done in the COnstraint-Based Reconstruction and Analysis (COBRA) Toolbox in MATLAB (Heirendt et al. 2019) using the Gurobi™ optimizer version 8.1.1 (Gurobi Optimization, LLC 2019).

### 2.5 Error evaluation

We assume that the ^13^C-MFA fluxes reported in Ma et al. (2014) are the true *in vivo* metabolic fluxes and therefore regard the discrepancy between FBA-predicted fluxes and these ^13^C-MFA fluxes as a measure of error. Biomass accumulation (i.e. the difference in dry weight between a timepoint *t* and another timepoint *t*_−1_) was not reported in Ma et al. (2014), but is the basis for the flux through the biomass equation in FBA. To allow a comparison between our FBA-predicted fluxes and the MFA-estimated fluxes in Ma et al. (2014), we set an arbitrary biomass flux of 0.01 g/h through the leaf, stem, and root biomass reactions in both the day and night, similar to the approach taken in de Oliveira Dal’Molin et al. (2015). We then normalized our fluxes by multiplying them by the ratio of the measured leaf CO_2_ uptake from Ma et al. (2014) and the net leaf CO_2_ uptake in our FBA flux map. A weighted average error for each FBA-predicted flux map was obtained using the following expression:


(7) $$\sum_{j\in Measured}\left(\frac{\left|{(v}_{j}^{p}*A)-v_{j}^{m}\right|}{\left|v_{j}^{m}\right|}*\frac{\left|v_{j}^{m}\right|}{\sum_{j\in Measured}\left|v_{j}^{m}\right|}\right)$$

where $v_{j}^{p}$ and $v_{j}^{m}$ are the FBA-predicted and MFA-estimated fluxes of a flux *j* and A is the normalization factor previously described. We calculated weighted average errors rather than just average errors because small absolute differences between FBA-predicted and MFA-estimated flux values can correspond to extremely large % error values when the MFA-estimated fluxes are small. We quantified the maximum/minimum weighted average errors of each flux map using flux variability analysis (FVA; Mahadevan and Schilling 2003). Additional details can be found in the Supplementary Methods.

## 3 Results

### 3.1 The application of gene expression penalty weights reliably reduces discrepancies between FBA-predicted and MFA-estimated fluxes

Predicted flux maps were generated for a multi-tissue diel model of *A.thaliana*’s central metabolism using FBA in which the sum of all the metabolic and transport fluxes required for steady-state growth is minimized, with each flux being multiplied by a penalty weight that was derived from the relative expression of the gene(s) involved in conducting that flux (see Section 2). Penalty weights for each reaction were calculated from RNA-seq (Klepikova et al. 2016; Mergner et al. 2020) and proteomic (Mergner et al. 2020) datasets using the relative expression of each gene in the different tissues. The weighted average % error between these flux maps and ^13^C-MFA estimates from Ma et al. (2014) were used to quantify the accuracy of these FBA predictions, as compared with the accuracy of flux maps generated by pFBA (Lewis et al. 2010) alone. The flux maps arrived at after the application of either transcriptomic or proteomic penalty weights show greater agreement, as measured by the weighted average % error, with ^13^C-MFA estimates than the results from pFBA alone (Table 1). These reductions in error are substantial and statistically significant at α = .01; they are consistent across comparisons against two different flux maps (high- and low-light conditions) and are sustained across a range of assumed ratios of starch to sucrose production and carboxylase to oxygenase fluxes through RuBisCO (vo/vc). Marked reductions in error are seen whether one uses the transcriptomic or proteomic tissue-atlas datasets from Mergner et al. (2020) or the transcriptomic dataset from Klepikova et al. (2016), so that the improvement in flux predictions is not dependent on the values obtained in a specific gene expression dataset or type.

**Table 1.** Weighted average % error values calculated from weighted versus unweighted flux maps for transcriptomic and proteomic datasets from Klepikova et al. (2016) and Mergner et al. (2020).^a^

Dataset	Light level	Weighted average error (%)	
		No gene expression weights	With penalty weights
Mergner et al. Transcriptome	High	169–180	14.7–17.1
	Low	93.8–103	14.9–18.1
Mergner et al. Proteome	High	169–180	10.9–13.4
	Low	93.8–103	8.74–10.9
Klepikova et al. Transcriptome	High	169–180	14.8–17.4
	Low	93.8–103	19.3–21.7

a Values represent the lowest and highest possible error values given the results of FVA. Weighted average error values were calculated from flux maps generated using a scaling factor of 1.

We wanted to confirm that these reductions in error are in fact dependent on penalty weights calculated from gene expression data and not an artifact of the weighting procedure itself. Indeed, previous studies have used the application of randomized weights as a method of exploring different possible flux modes in a plant metabolic network (Cheung et al. 2015). We found that substituting the leaf for the root proteomic dataset, and vice-versa, resulted in no reduction in weighted average error (Supplemental Table S1) compared with pFBA. Neither did randomization of the penalty weight vector and subsequent optimization. The mean of the weighted average errors of 50 high-light condition flux maps generated with independent randomized penalty weight vectors at a scaling factor of 1 was 201%, versus the unweighted error value of 169%–180% for that condition.

### 3.2 Increases in agreement between FBA-predicted and MFA-estimated fluxes are broadly distributed across central metabolism

**Figure 1:**
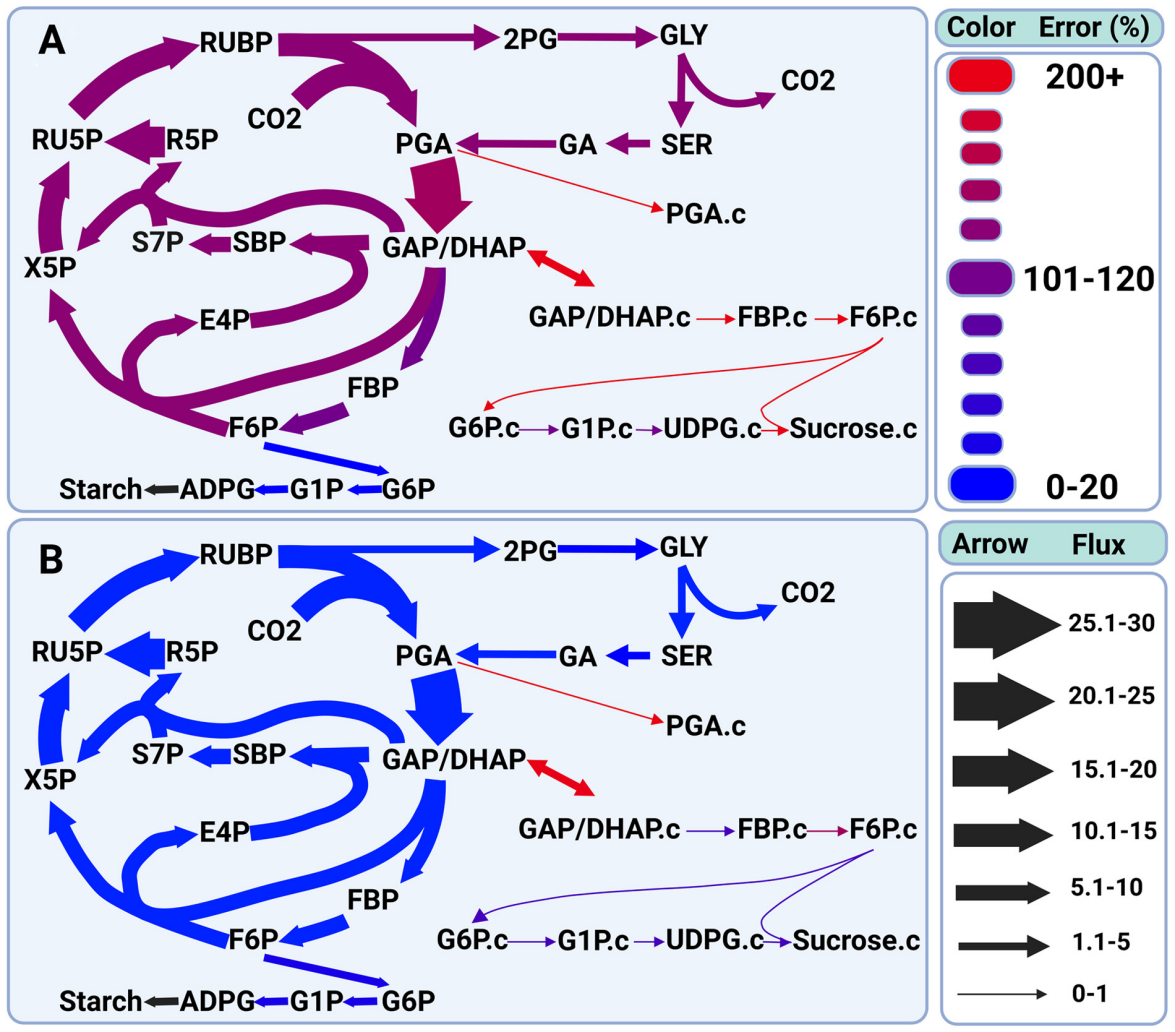
Percent errors of specific reactions in central metabolism before (A) and after (B) gene expression weight application. The error values in (A) are the lowest possible given FVA results and the values in (B) are the highest possible given FVA results. We see substantial decreases in errors associated with central carbon assimilation, as well as starch and sucrose synthesis. Since the ^13^C-MFA estimated fluxes from Ma et al. (2014) do not feature the flux from ADPG to Starch, this flux lacks an estimated error and is therefore shown in black. Flux values are relative to the lowest flux in the network.

Although there is variation among individual fluxes in the degree to which omic data integration improves agreement between predicted and experimentally derived values, the reduction in weighted error as a result of penalty weight application is distributed broadly across the fluxes for which ^13^C-MFA estimates are available. If, for example, the improvement were due to a substantial decrease in one or a small number of high-flux reactions and a negligible decrease or even increase in error for other reactions (Fig. 1) the overall finding would be less striking and potentially less broadly applicable. The reductions in error are consistent not only across metabolic subsystems within a single FBA flux map, but also across alternative stoichiometric network structures. Initial pFBA-derived solutions for a model identical to that used to generate the other predictions except with unconstrained uptake and discharge of protons from root tissue show similar reductions in error (Supplementary Table S2). Upon application of penalty weights, this model converges to a similar value of weighted average error and linear correlation as other model configurations.

### 3.3 Error reductions are a function of the scaling factor parameter and are improved by the application of a tissue-specific median weight for reactions lacking gene protein reaction terms

**Figure 2:**
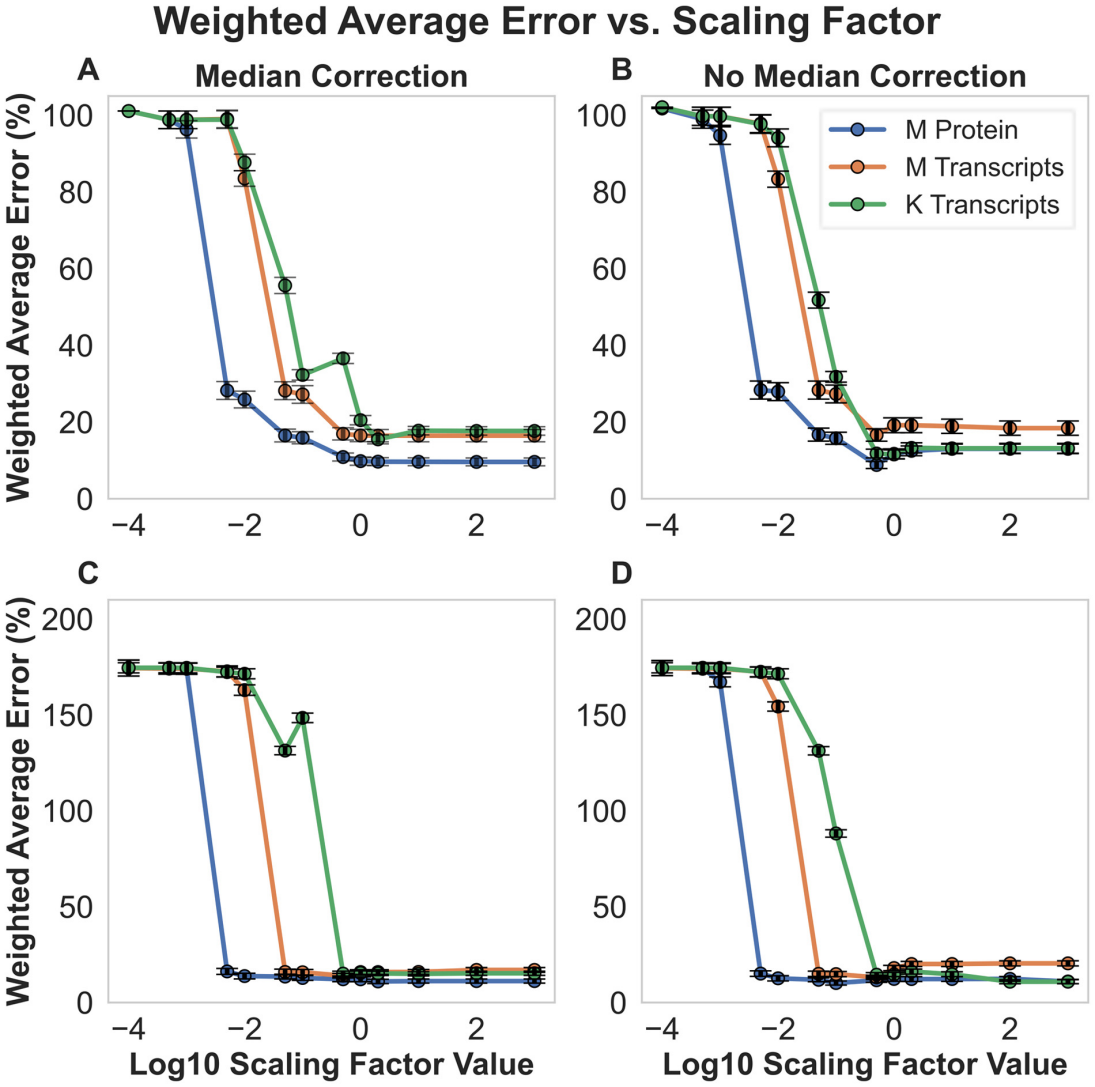
Weighted average errors of FBA predictions compared with MFA-estimated flux maps as a function of scaling factor value, light-level, and application of a tissue-specific median weight correction. Panels show weighted average errors of flux maps generated using (A) low-light constraints and a tissue-specific median correction applied, (B) low-light constraints and without a tissue-specific median correction applied, (C) high-light constraints and with a tissue-specific median correction applied, and (D) high-light constraints and without a tissue-specific median correction applied. “M Protein” and “M Transcripts” refer to flux maps generated using proteomic- and RNA-seq-derived weights from Mergner et al. (2020). “K Transcripts” refers to flux maps generated using RNA-seq derived weights from Klepikova et al. (2016). Upper and lower bars on each point represent the highest and lowest possible weighted average errors given FVA results, and the points themselves represent the average of these values.

The magnitude of the penalty weights calculated and applied by the present method depends on the magnitude of the scaling factor term, (Equation 3). The increased agreement between the FBA-predicted and MFA-estimated flux maps only manifests in the majority of cases for scaling factors of 0.05–0.1 or greater (Fig. 2). We also note that the relationship between the scaling factor value and the improved agreement is monotonic—i.e. we do not see erratic increases and decreases as we increase the scaling factor value and, by extension, the strength of the assumed relationship between flux and gene expression. The necessity of a non-negligible scaling factor, the consistency of error improvement as the scaling factor is increased, and the similarity in the pattern of error improvement across multiple datasets as seen in Fig. 2, all suggest that real biological signal related to the partitioning of metabolic activity across the plant’s tissues is being extracted from the gene expression datasets. Finally, we observe that the flux maps generated using penalty weight derived from Mergner et al. (2020) proteomic dataset have noticeably better weighted average errors than flux maps generated using transcriptomic dataset (Table 1 and Fig. 2). This is consistent with the closer relationship between measured protein levels and metabolic fluxes than between transcripts and fluxes. It is also consistent with at least one other study’s attempts at integrating gene expression data into FBA in *E.coli* (Tian and Reed 2018).

Although the method presented does not involve fitting the Scaling Factor parameter using goodness-of-fit to the 13C-MFA fluxes, in Fig. 1 and Tables 1 and 2, we show results from a Scaling Factor of 1 because it falls in the plateau of low average error values we see in Fig. 2. To further explore the usefulness of a Scaling Factor of 1, we used the fluxes reported in Szecowka et al. (2013) for illuminated *A.thaliana* leaves estimated by kinetic flux profiling. The FBA-derived flux map generated using *vo/vc* and starch: sucrose synthesis constraints from that study without any omic weighting has a weighted average error of 108%; this error drops to 8, 6, and 9% when protein or transcript weights from Mergner et al. (2020) or transcript weights from Klepikova et al. (2016), respectively, are applied with a Scaling Factor of 1 (Supplementary Table S6 and Dataset S5).

In our initial formulation of the algorithm for generating gene expression-derived penalty weights, the weight of all reactions with no associated GPR was set to 1, since this is the implicit value of the coefficient for all reactions in a standard pFBA optimization. Since this runs the risk of introducing a systematic bias against using reactions that have associated GPRs, we attempted to counteract this risk by assigning all reactions lacking a GPR a penalty weight corresponding to the median penalty weight of all weighted reactions in the tissue in which those reactions are found. Comparing the results with and without the tissue-specific median penalty weights for reactions without GPRs, we see modest improvements in the weighted average errors from a scaling factor of 1 onwards when using the transcriptomic and proteomic datasets from Mergner et al. (2020; Fig. 2), though the effect is not large, indicating that our method is robust to including or omitting the tissue-specific median weight correction.

### 3.4 Changes in the carbon and energy economy upon application of gene expression weights

In addition to improving quantitative agreement between the FBA-predicted and MFA-estimated flux maps, the gene expression weighting procedure also generates flux maps that present a substantially different picture of carbon and energy metabolism in *Arabidopsis* leaves.

In both high and low light FBA-predicted fluxes there is a substantial decrease in leaf mitochondrial electron transport chain (ETC) activity and overall flux in mitochondria-localized reactions in the light relative to nighttime ETC activity and overall flux (Supplementary Table S3). MFA and other recent work further points to low TCA cycle fluxes in photosynthesizing leaves (Tcherkez et al. 2005; Xu et al. 2021; 2022). This decrease in mitochondrial activity goes hand-in-hand with a predicted decrease in the use of unusually high fluxes related to proline metabolism to indirectly support the consumption of excess reductant produced via the light reactions of photosynthesis. Alongside this decrease in mitochondrial activity is a decrease in the ratio of cyclic electron flow (CEF) to linear electron flow (LEF) in the chloroplast (Table 2). Although reliable empirical measurements of this CEF/LEF ratio are difficult to obtain, previous studies have shown that a C3 plant like *Arabidopsis* relying on CEF to bring the ratio of ATP/NADPH produced up to that needed for normal growth would have a CEF amounting to ∼13% of LEF (Kramer et al. 2004). Due to the presence of other balancing mechanisms, such as the malate valve (Selinski and Scheibe 2019), this 13% value would represent an upper bound on stoichiometrically predicted values for CEF/LEF. Application of gene expression data decreases the CEF/LEF ratios in all but one FBA-predicted flux map to values much closer to the expected ∼13% upper bound than are predicted using conventional pFBA (Table 2).

**Table 2.** Measures of carbon and energy utilization derived from the predicted flux maps with and without penalty weights applied.

Dataset used for weighting	Light	RuBisCO flux ÷ net CO_2_ assimilation	Photorespiratory CO_2_ loss/net CO_2_ assimilation (%)	CEF/LEF (%)	% of leaf daytime CO_2_ assimilation going to biomass
None	High	2.86	62	24	43
	Low	1.85	26	31	54
Mergner *et al.* Protein	High	1.29	26	20	18
	Low	1.17	14	15	26
Mergner *et al.* Transcripts	High	1.20	25	21	18
	Low	1.15	14	27	33
Klepikova *et al.* Transcripts	High	1.30	27	17	19
	Low	1.25	15	14	31
Reference values	High	1.28^a^	28^a^	13^b^	56%^c^
	Low	1.17^a^	16^a^		

a Ma et al. (2014).

b Kramer et al. (2004).

c Weraduwage et al. (2015). The superscript letters b and c reference values are not associated with a particular light level.

Ma et al. (2014) reported MFA-derived estimates of %vpr, or the rate of photorespiratory CO_2_ release via glyoxylate decarboxylation as a % of CO_2_ assimilation, as well as the ratio of RuBisCO carboxylation flux to net CO_2_ assimilation in the leaf. The unweighted flux predictions for the high and low light conditions disagree substantially with these estimates (Table 2). However, the application of gene expression weights consistently brings estimates of these parameters into close agreement with MFA-derived values. The integration of gene expression also changes the predicted efficiency with which *Arabidopsis* converts atmospheric CO_2_ into biomass (Table 2). For comparison with these predicted efficiencies, we used the empirical *A.thaliana* biomass, leaf area, and gas exchange data reported in Weraduwage et al. (2015) to calculate that ∼56% of the net CO_2_ assimilation in illuminated leaves ends up in incorporated into biomass, which is closer to the value in our unweighted flux predictions than our weighted flux predictions, although it should be noted that these data were gathered from a hydroponic system.

## 4 Discussion

^13^C-MFA is broadly accepted as being the most reliable method for estimating metabolic flux maps *in vivo* due to its ability to make use of substantial amounts of isotopic labeling data to arrive at well-supported flux maps in small- to medium-scale networks (Antoniewicz 2015). However, the technique’s utility is limited by the substantial experimental effort that goes into the generation of each individual flux map. FBA, with its requirement of much less experimental data, has become the method of choice for more exploratory or predictive metabolic modeling studies. The implicit assumption is usually that the predictions of FBA—or at least the range of its predictions in cases where a unique solution is not provided—agree with those we would arrive at if we were able to conduct a ^13^C-MFA study. This makes our optimization procedures when performing FBA and validation of FBA models against MFA results of vital importance. The method presented here, by bringing FBA-predicted fluxes into line with MFA estimates represents a step in the direction of higher-confidence FBA flux maps.

One limitation, as well as motivation, for this study is the lack of a large set of ^13^C-MFA datasets in plants and other multi-tissue eukaryotic systems. Systems like *E.coli* have multiomic datasets consisting of transcriptomic, proteomic, and fluxomic measurements (Ishii et al. 2007) that have been utilized to empirically infer the relationship between gene expression and metabolic fluxes. This empirical training can then be used to more accurately predict fluxes in new contexts (Tian and Reed 2018). The sparsity of ^13^C-MFA data in more complex systems makes such an approach currently impossible.

A noteworthy theoretical aspect of the present approach is its simplicity, the only variable parameter being a single scaling factor that controls the magnitude of the penalty weights. That the assumption of a consistent value relating the relative abundances of transcripts or proteins in different tissues to the “preference” of an organism to partition flux among particular reactions can result in substantial improvement in error was of great interest in light of the complexity of the relationship between measures of gene expression—transcriptomic and proteomic abundances—and flux. Particularly when making biotechnological interventions in a system to modify its metabolism, there is often an assumed strong linear relationship between transcription, translation, and, ultimately, metabolic flux, but the reality is rarely so simple. Although moderate correlations between transcript and protein abundances have been demonstrated across many systems, the degree of correlation varies across systems and experimental contexts (Maier et al. 2009; Liu et al. 2016). The correlation between these data types and rates of central metabolic reactions, which carry the large majority of total metabolic flux, is weaker still (Kuile and Westerhoff 2001). Some previous studies found that changes in the gene expression related to individual reactions typically do not correlate well with changes in fluxes (Schwender et al. 2014; Tian and Reed 2018), with some central metabolic fluxes in particular showing a negative correlation between changes in gene expression and flux. In both cases, gene expression data related to reactions were compared within the same cell type or tissue; in our study, we instead compare intertissue abundances, mirroring the long-standing practice in the literature of inferring relative metabolic activity in different tissues by their transcript and protein investment in relevant pathway steps. It may be that only by considering gene expression on an intertissue basis in the context of the entire complex stoichiometric network underlying metabolism can predictive gains from including gene expression evidence be properly realized.

Future work should aim to expand the number of available datasets, and the experimental conditions and genotypes for which they are gathered, in order to enable more thorough evaluation of methods like the one presented in this article. Indeed, evaluating the presented method requires ^13^C-MFA fluxes, multi-tissue omic data, and a GEM all for the same biological system, which, to our knowledge, is currently only available for *A.thaliana*. Building on the work of Ma et al. (2014), experimental improvements and refinements of the underlying network architecture of central carbon metabolism have been introduced in the context of ^13^C-MFA in *Camelina sativa* (Xu et al. 2021; 2022) and *Nicotiana tabacum* (Chu et al. 2022). In this study, Ma et al. (2014) flux maps are used without change and we adopted a highly curated *A.thaliana* GEM from which to construct the whole-plant model. This approach precluded the possibility of our reanalyzing the MFA-estimated flux map or biasing the construction of a purpose-built GEM, making the MFA-to-FBA comparison more favorable. However, in future studies, a combination of MFA network refinements, expanded datasets, and further improvements in the flux estimation procedures holds promise for improving the fidelity of the ^13^C-MFA comparison data. On the FBA side, the use of more detailed growth and composition measurements for FBA along with more detailed representation of different tissue types will potentially allow for more biologically accurate and representative FBA flux map predictions. These improvements in both MFA-estimation and FBA-prediction of flux maps, along with an expansion in the number of available ^13^C-MFA datasets against which to compare FBA predictions, will allow for more extensive validation of the method described in this paper as well as other methods aiming to incorporate omic datasets into flux prediction.

A distinct aspect of the proposed method is its demonstrated ability to bring FBA-predicted fluxes in line with MFA-estimated fluxes across multiple input datasets, model architectures, and using multiple independent gene expression datasets. Our hope is that methods for incorporating transcriptomic and proteomic data may advance this field to the point where FBA-predicted flux maps can be used with high confidence for practical engineering goals. This, combined with the automated reconstruction of GEMs from genomic and biochemical databases (Saha et al. 2014) suggests a future with rapid turnaround from the initial identification of an organism of interest to metabolic flux predictions and rational genetic engineering to achieve biotechnological aims.

## Supplementary Material

btad186_Supplementary_Data
